# Low serum adiponectin concentrations are associated with insulin sensitivity independent of obesity in Sudanese subjects with type 2 diabetes mellitus

**DOI:** 10.1186/1758-5996-5-15

**Published:** 2013-03-13

**Authors:** Moawia Abdelgadir, Anders F Karlsson, Lars Berglund, Christian Berne

**Affiliations:** 1 Department of Medical Sciences, Uppsala University Hospital, Uppsala SE.751 85, Sweden; 2 Uppsala Clinical Research Centre, Uppsala University Hospital, Uppsala, Sweden

**Keywords:** Diabetes mellitus, Sudan, Adiponectin, Ghrelin, Leptin

## Abstract

**Aims:**

Prevalence of Type 2 diabetes mellitus among Sudanese population was found to be 3.4% and associated with high rates of complications and obesity. Different adipocytokines are secreted from adipose tissues, among them adiponectin, which was shown to have insulins ensitizing properties and anti-inflammatory, anti-atherogenic effect. The aim of this study was to characterize type 2 diabetes in Sudanese diabetic subjects and controls in respect to hormones influencing or influenced by glucose metabolism.

**Methods:**

104 type 2 diabetic patients (45 men and 59 women), and 75 matched control subjects (34 men and 41 women) were studied. Fasting serum samples were used to measure adiponectin, leptin, insulin, proinsulin, ghrelin and glucose. Body mass index, insulin/proinsulin ratio and (HOMA) insulin resistance and beta cell function were also calculated.

**Results:**

Adiponectin serum concentrations were significantly lower in subjects with type 2 diabetes compared with controls subjects (P = 0.002), comparison between males and females did not reach significant levels in both diabetic (P = 0.06) or controls (P = 0.16) groups. In the diabetic group adiponectin correlated positively with serum glucose, negatively with serum proinsulin and HOMA beta cell function (P = 0.03) respectively and serum ghrelin (P = 0.003), but not with BMI, HOMA insulin resistance, insulin or leptin. In controls serum adiponectin correlated negatively with BMI (P = 0.002) but not with other variables.

**Conclusions:**

The findings of this study suggest that, adiponectin concentrations independent on BMI as a measure of adiposity, were mostly linked to insulin sensitivity and not to insulin resistance in Sudanese type 2 diabetic subjects, where race specific regulation mechanisms or different type 2 diabetes phenotype suggested being a major contributory factor in clarification the findings of this study.

## Introduction

Impaired insulin secretion or/and abnormal insulin action and obesity are major characteristics for type 2 diabetes mellitus [1-3]. The prevalence of diabetes mellitus in the Sudanese population was 3.4% and type 2 diabetes mellitus account for 75% of all diagnosed cases, among whom the majority also has a family history of diabetes [4] and 40% of them are obese [5] In a population based study 40% of the patients are found to be obese [5].

Adipose tissue was found to produce a variety of adipocytokines including leptin, adipsin and tumor necrosis factor [6-11]. Adiponectin is the recently identified most abundant among of them – is a 30. kDa protein [12-14]. Mechanisms of regulation of adiponectin proposed to be multifactorial. Involvements of genetic factors, glucocorticosteroids, body fat distribution and insulin have been shown in different studies [12,15,16]. Adiponectin serum levels were found to be lower in type 2 diabetes mellitus and obese subjects [14,17-19] and reversibly correlated with insulin resistance and measures of body fat [20-23]. Besides its insulin.sensitizing properties [23-25], adiponectin was also suggested to have an anti-inflammatory, anti-atherogenic effect [26-28].

It is of interest to explore the association of adiponectin with metabolic parameters in different populations where ethnic diffrences were reported in som studies [29-34]. The aim of this study was to characterize type 2 diabetes in Sudanese diabetic subjects and controls in respect to hormones influencing or influenced by glucose metabolism.

### Research design and methods

One hundred and four diabetic patients (males/females, 45/59) were recruited from the outpatient diabetes clinic at Omdurman Teaching Hospital, Khartoum, Sudan. Inclusion criteria were treatment with diet and/or oral hypoglycemic agents, age > 20 years and duration of diabetes >1 year. They were treated with glibenclamide (n = 80), glicalazide (n = 1), metformin (n = 4) and or diet alone (n = 19). A BMI and age matched group of 75 (males/females, 41/34) apparently healthy non diabetic subjects, who live in the same area, were selected as controls. Informed consent was obtained from all subjects. The and ethical clearance was taken from committee at The Federal Ministry of Health approved of the study. A questionnaire including personal details and clinical characteristics was completed for all subjects. Blood pressure, weight and height in light clothing without shoes were measured and body mass index (BMI) was calculated. In the fasting state blood samples were drawn for the determination of serum adiponectin, leptin, ghrelin, glucose, insulin and proinsulin. The samples were centrifuged within 2 hours after collection and kept frozen at −20°C until analyzed at the Department of Medicine, Malmö University Hospital and Uppsala University Hospital, Sweden. The demographic and clinical characteristics of the subjects are shown in Table 1.

**Table 1:** Demographic and clinical characteristics of the subjects

**Characteristics**	**Type 2 diabetes**	**Controls**
No. of subjects	104	75
Males/Females	45/59	34/41
Age (yrs)	53.8 ±10.7	53.4 ±8.9 *(P = 0.83)*
BMI (kg/m2)	23.5 ± 5.3	24.0 ±3.7 *(P = 0.59)*
Diabetes duration (yrs)	9.9 ± 7.9	-
Systolic BP (mmHg)	117 ±14	119 ±16.0 *(P = 0.22)*
Diastolic BP (mmHg)	79 ± 8	80 ± 10 (*P = 0.71)*
Adiponectin (μg/ml)	7.54 ± 4.62	9.56 ± 5.92 *(P = 0.0017)*
Gherlin (μg/ml)	26.78 ± 18.91	26.64 ± 8.78 *(P = 0.99)*
Leptin (ng/mL)	9.6 ± 7.9	14.9 ± 11.6 *(P = 0.0066)*
Glucose (mmol/L)	8.6 ± 4.4	4.2 ± 0.9 *(P = 0.0001)*
Insulin (pmol/L)	75 ± 47.0	67.6 ± 23.2 *(P = 0.99)*
Proinsulin (pmol/L)	26.3 ± 21.7	17.8 ± 12.8 *(P = 0.0031)*
PI/insulin ratio	0.38 ± 0.30	0.24. ± 0.12 *(P = 0.0003)*
HOMA IR	6.15 ± 0.92	5.58 ± 0.40 *(P = 0.0001)*
HOMA B	2.82 ± 1.26	4.62 ± 0.90 *(P = 0.0001)*

Mean ± SD Abbreviations: *BP*, blood pressure; *PI*, proinsulin.

Adiponectin was analyzed using Human Adiponectin RIA kit (Linco Research, St. Charles, MO, USA) of assay specificity <0.01%, ghrelin was measured using Human Ghrelin RIA kit (Linco Research, St. Charles, MO, USA) of 100% specificity and leptin was measured using RIA kit (Linco Research, St. Charles, MO, USA), detecting immunoreactive human leptin with sensitivity 0,5 ng/mL. Insulin was measured with specific RIA cross reacting with less than 0.2% proinsulin (Linko Research, St. Charles, MO, USA). Total serum proinsulin levels were measured using a goat antibody raised against human proinsulin, human proinsulin standards and 125I-human proinsulin as tracer (Linco Res. Inc.). This assay detects intact proinsulin (100%) and des.31,32 proinsulin (95%) but does not crossreact with insulin, C-peptide or des-64,65 proinsulin (<0.1%). Serum glucose was analyzed using the glucose oxidase technique. Homeostasis model assessment (HOMA) was used to assess pancreatic cell function (HOMA B) and insulin resistance (HOMA IR) using fasting insulin and glucose concentrations by the formula: HOMA-B (%) =20 X [insulin]/(glucose.3,5) and HOMA-IR = insulin/(22,5e.ln[glucose]) [35].

### Statistical analysis

All data were expressed as mean + SD. Statistical analysis was performed using the program SAS for Windows 6.12 (SAS Institute, Cary NC). *T*-test was used for comparison between groups for variables with normal or log normal distribution. Pearson’s correlation coefficient was used to determine association with variables normal or log normal distribution. Adjustment calculated as Pearson’s partial correlation coefficients.

## Results

Table 1 shows the characteristics of the two study groups. Fasting adiponectin serum levels were significantly lower in diabetic subjects compared to controls (Table 1). However, differences did not reach significance when males and females in both diabetic 6.9 ± 3.5 vs. 8.4 ± 5.2 μg/ml, (P = 0.06) and control 9.1 ± 6.3 vs. 10.2 ± 5.4 μg/ml, (P = 0.16) groups where compared. Adiponectin did not correlate to BMI in diabetic group(r = −0.12, P = 0.24), but it correlated significantly negative in the control subjects (r = −0.38, P = 0.002).

Adiponectin concentrations correlated significantly negative with HOMA B in subjects with type 2 diabetes only (r = −0.21, P = 0.04), whereas no significant correlation to HOMA IR was found in subjects with type 2 diabetes (r = 0.08, P = 0.44) or controls (r = −0.16, P = 0.33). Serum adiponectin in diabetic subjects did not correlate significantly with systolic (r = −0.13, P = 0.20) and diastolic (r = −0.13, P = 0.20) blood pressure, this correlation remained non-significant after adjustment for BMI. In non-diabetic subjects, the systolic and diastolic blood pressures were not related to adiponectin concentrations.

Adiponectin levels correlated significantly negative to serum proinsulin in diabetic subjects (r = −0.22, P = 0.03), but not to insulin (r = −0.16, P = 0.12) or proinsulin/insulin ratio (r = −0.18, P = 0.06), whereas no correlation was seen between adiponectin and the insulin or proinsulin/insulin ratio in control subjects (r = 0.03, P = 0.83) and (r = −0.01, P = 0.53).

Also serum adiponectin levels correlated significantly positive with serum glucose in diabetic subjects (r = 0.22, P = 0.03), however no significance was found between adiponectin and serum glucose in control subjects (r = −0.1, P = 0.5).

Type 2 diabetic subjects treated by sulphonylurea, had almost the same levels of adiponectin (7.3 ± 4.6 μg/ml, n = 81) compared to those treated with diet alone, or metformin (7.6 ± 4.7 μg/ml, n = 23, P = 0.99). Adjusting for BMI (P = 0.87) did not changed the results. The subjects treated with sulphonylurea had, however, also lower BMI than the other group, (22.7 ± 4.9 versus 26.1 ± 5.9, P = 0.0017), but no differences in HOMA B (P = 0.28) or HOMA IR (P = 0.15) was found. Fasting serum leptin levels did not correlate with adiponectin levels neither in diabetic (r = 0.03, P = 0.73) nor in control (r = −0.01, P = 0.1) groups.

However, serum adiponectin level correlated significantly with serum ghrelin in diabetic subjects (r = 0.29, P = 0.003), but not in control subjects (r = 0.18, P = 0.14).

## Discussion

In agreement with previous reports adiponectin was found to be significantly lower in subjects with diabetes compared to controls [17-19]. However, in contrast with the studies that have shown that irrespective of ethnicity, adiponectin concentrations negatively correlated to BMI and insulin resistance in subjects with diabetes [14,17,18,23]. Our findings showed that adiponectin in diabetic subjects correlated positively with fasting glucose and negatively with fasting serum proinsulin and HOMA cell function but not with the insulin or HOMA insulin resistance or even BMI. In the control subjects adiponectin correlated negatively only with BMI. The difference in adiponectin concentrations and its association in both groups could be explained by the fact that adiponectin ameliorates insulin sensitivity, where healthy controls have significantly increased adiponectin levels, thus have significantly lower insulin resistance and better cell function (Table 1), and that goes in agreement with some studies [36]. However, the finding in this study that showed similar adiponectin levels in two diabetic subgroups (according to type of treatment) that have significantly different BMI, and have no differences in HOMA IR or HOMA B, could confirm the finding that adiponectin concentrations are linked also to insulin sensitivity [24,25] irrespective to measures of adiposity [36]. Relaying on the findings that adiponectin related to proinsulin and HOMA cell function in diabetic subjects, it would be useful to find whether there is a possible role of adiponectin in mediation hepatic IR and decreased cell function in type 2 diabetes, as has been recently demonstrated that the interaction between muscle and hepatic receptors through activation of AMP kinase could mediate adiponectin effects [37].

Besides, due to differences in fat distribution, as diabetics have less subcutaneous fat and increased visceral fat, lipotoxic effects could lead also to deterioration of pancreatic cell function and increased hepatic and peripheral insulin resistance in type 2 diabetes [38].

In both groups we did not observe significant gender differences in adiponectin concentrations [39]. Also no association was found between adiponectin and leptin and that is similar to a previous report [40]. Data on association between adiponectin and ghrelin in diabetes is scarce, and no correlation has been found in non diabetic subjects [41,42]. Serum adiponectin and ghrelin concentrations were found to be significantly correlated only in subjects with diabetes in this study, where ghrelin correlated significantly negative with fasting glucose in both diabetic (r = 0.21, P = 0.03) and control subjects (r = 0.40, P = 0.008). The link between adiponectin and ghrelin in diabetic subjects could be through the hepatic glucose homeostasis, which is enhanced by ghrelin and suppressed by adiponectin [43]. Despite the generally known association between adiponectin and some metabolic and adiposity characteristics, the absence of this relationship as shown in this study, could support the hypothesis that adiponectin concentration could be regulated differently in different populations, particularly in populations with an African origin [29-34].

In conclusion, we showed in this study different association of adiponectin in Sudanese diabetic and healthy subjects, contrasting to other reports of different populations.

Adiponectin concentrations are mostly linked to insulin sensitivity independent on BMI as a measure of adiposity and not to insulin resistance in type 2 diabetic Sudanese subjects. Also adiponectin concentrations were linked to ghrelin levels in subjects with type 2 diabetes. However, race specific regulation mechanisms or a different type 2 diabetes phenotype could be factors in clarification the findings of this study. Further investigations among this population and population oriented approach to elucidate the mechanisms underlying these perturbations of adiponectin concentrations are required.

## Competing interests

The authors declare that they have no competing interests.

## Authors’ contributions

(MA, AFK, LB and CB) contributed equally to this work. All authors read and approved the final manuscript.
